# Cell Pluripotency Levels Associated with Imprinted Genes in Human

**DOI:** 10.1155/2015/471076

**Published:** 2015-10-04

**Authors:** Liyun Yuan, Xiaoyan Tang, Binyan Zhang, Guohui Ding

**Affiliations:** ^1^Key Laboratory of Systems Biology, Institute of Biochemistry and Cell Biology, SIBS, CAS, Shanghai 200031, China; ^2^BasePair BioTechonology Co., Ltd., Shanghai 200235, China; ^3^Shanghai Center for Bioinformation Technology, Shanghai 200235, China

## Abstract

Pluripotent stem cells are exhibited similarly in the morphology, gene expression, growth properties, and epigenetic modification with embryonic stem cells (ESCs). However, it is still controversial that the pluripotency of induced pluripotent stem cell (iPSC) is much inferior to ESC, and the differentiation capacity of iPSC and ESC can also be separated by transcriptome and epigenetics. miRNAs, which act in posttranscriptional regulation of gene expression and are involved in many basic cellular processes, may reveal the answer. In this paper, we focused on identifying the hidden relationship between miRNAs and imprinted genes in cell pluripotency. Total miRNA expression patterns in iPSC and ES cells were comprehensively analysed and linked with human imprinted genes, which show a global picture of their potential function in pluripotent level. A new CPA4-KLF14 region which locates in chromosomal homologous segments (CHSs) within mammals and include both imprinted genes and significantly expressed miRNAs was first identified. Molecular network analysis showed genes interacted with imprinted genes closely and enriched in modules such as cancer, cell death and survival, and tumor morphology. This imprinted region may provide a new look for those who are interested in cell pluripotency of hiPSCs and hESCs.

## 1. Background

Undifferentiated embryonic stem cells (ESCs) share the ability to self-renew and differentiate into various different lineages which is fundamental to understanding human development, tissue regeneration, and healthy homeostatic turnover [1, 2]. It has been demonstrated that, only by addition of a few defined factors, pluripotent stem cells can be directly generated from fibroblast cultures [3, 4] and are exhibited similarly in morphology, gene expression, growth properties, and epigenetic modification as ESCs [5, 6]. The reprogramming techniques open eyes for understanding the developmental mechanisms in assigning cells for particular fates and are also bound to provide us with questions about the different pluripotency levels. The pluripotency of induced pluripotent stem cell (iPSC) is much inferior to ESCs, as can be separated by transcriptome and epigenetics. Several recent studies focused on identifying the hidden difference between ES and iPSCs and tried to break the block on the progress of its basic research and clinical application [7, 8].

MicroRNAs (miRNAs) are a class of noncoding RNA genes whose products are 22nt sequences that play significant roles in the regulation of translation and degradation of mRNAs through base pairing to partially complementary sites in the untranslated regions (UTRs) of the message [9]. miRNAs were intensely investigated to identify their mechanisms of action in cell development and progression [10–12]. Studies have reported that several miRNAs expressed diversely in iPSC and hESC have great effect on pluripotency [13, 14]. Interestingly, through analysing the small RNA expression patterns in iPSC, a Dlk1-Dio3 region with a large cluster of miRNAs as well as imprinted genes was identified in fully pluripotent stem cells [8]. This imprinted genomic region was found to be repressed in the cells with partial pluripotency, and aberrant silencing of this region in mouse was found to induce pluripotent stem cells. Mammals genomic imprinting is possibly caused by an interparental genetic conflict to control maternal-dependent growth of the offspring [15] and was found to be linked to a number of human behavioral and developmental disorders as well as a variety of pediatric and adult malignancies [16]. While imprinted genes were also found near differentially expressed miRNAs in hESCs [8, 17], these phenomena suggested that miRNAs may influence early development together with imprinted genes. In order to scan the association between miRNAs and imprinted genes in regulating cell pluripotency levels, we focus on miRNA expression profiles in both ESC and iPSC. miRNA clusters were found near imprinted genes on most chromosomes and an imprinted region which we think may influence hESCs pluripotency capacity will be discussed in the following paragraph.

## 2. Results and Discussion

### 2.1. miRNA Expression Analysis in Cells with Diverse Pluripotent Levels

**Figure 1:**
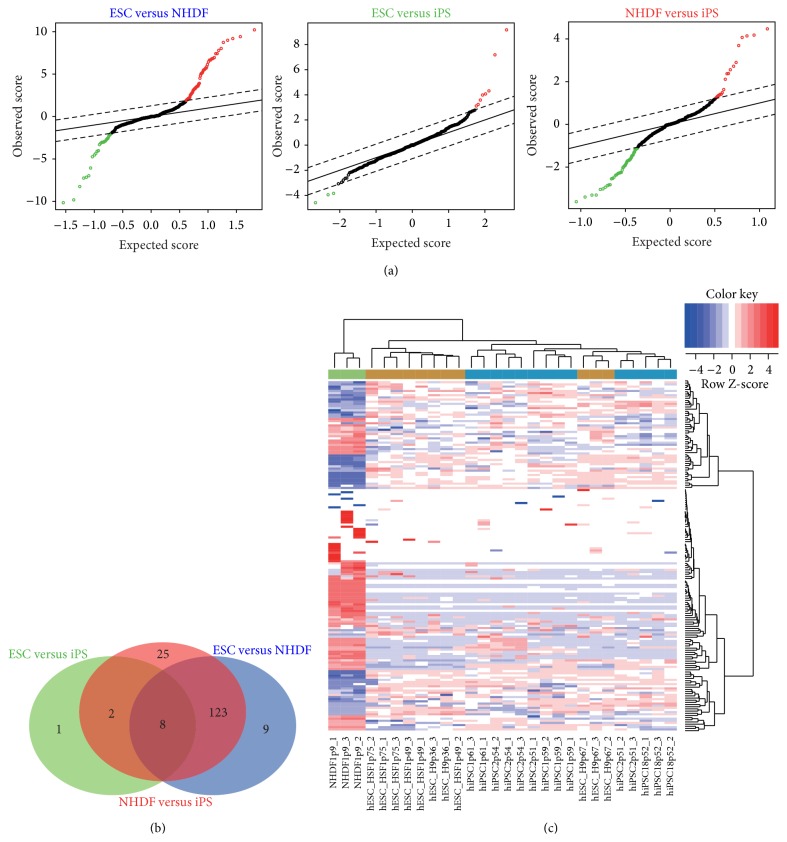
miRNAs expression profiles in different cells. (a) Significance analysis of microarray (SAM) plot of differentially expressed miRNA in hESCs (12), NHDF (3), and hiPSCs (15). The central solid line indicates equal expression and the upper and lower dashed lines indicate levels for significantly altered expression where FDR is 0%. (b) Venn diagram of differentially expressed miRNAs from pairwise comparisons, respectively. miRNA groups are coloured according to different comparison (green = ESC versus iPS; red = NHDF versus iPS; blue = ESC versus NHDF). (c) Heatmap showing hierarchical clustering of significantly regulated microRNA from iPS and ESCs compared with NHDFs. Blue indicates low expression and red indicates high expression.

We analysed a miRNA expression dataset stored in the GEO database which includes 800 miRNAs from normal human dermal fibroblasts (NHDFs), hiPSCs, and hESCs [18] (Section 4). Linear model [19] was implemented in pairwise comparison between hESCs and hiPSCs, NHDFs and hiPSCs, and hESCs and NHDFs, respectively (Figure 1(a)), by Limma package of Bioconductor. After normalization and statistical analysis, 174 miRNAs were identified and differentially expressed between pairwise comparisons (FDR *P* ≤ 0.05) (Figures 1(b) and 1(c)) and 131 miRNAs were significantly regulated in both hiPSCs and hESCs compared to NHDFs, among them, 79 with at least 2-fold change were considered to be directly related to cell pluripotency and may dominate during individual development progress (see Supplementary Table 1 in Supplementary Material available online at http://dx.doi.org/10.1155/2015/471076). These results included 14q32 microRNA clusters neighbouring the DLK1-DIO3 imprinted regions as well as those miRNAs in pluripotency, reprogramming, and cell fate induction [13, 20]. Besides, 40 miRNAs were found to be significantly regulated in hiPSCs (or hESCs) and slightly in hESCs (or hiPSCs) compared to NHDFs. These miRNAs may influence pluripotency levels between hESCs and hiPSCs.

To see the biological function of these differentially expressed miRNAs, miRNA targets were predicted to examine whether they were associated with cell pluripotency. The miRanda (release 2010) [21] and TargetScan (release 6.2) [22] lists were combined to form an intersection of targets predicted by both algorithms to strengthen the analysis reliability. Selected genes were then collectively subjected to pathway analysis using the R package. The enriched pathways are relevant to neural connectivity and synaptic plasticity, such as axon guidance, long-term potentiation, neurotrophin signaling pathway, and calcium signalling pathway (Supplementary Table 2). The results also show relevance to carcinogenesis including prostate cancer, pancreatic cancer, and pancreatic cancer, which is directly related to cell development and proliferation. The GO enrichment analysis was also performed as shown in Supplementary Table 2.

### 2.2. Differentially Expressed miRNAs Clusters Overlapped with Imprinted Gene Regions

In order to identify the association between the 131 differentially expressed miRNAs and imprinted genes, PTMCluster algorithm [23] was used to find the statistically significant miRNA clusters (Section 4). 12 differentially expressed clusters including 65 miRNAs were located on chromosomes, the length of which differed from 483bp (chromosome 4) to 111Mb (chromosome 1). Human imprinted genes were collected from Geneimprint website (http://www.geneimprint.com/) and totally 30 of them fall in or near the above miRNA clustering regions. To demonstrate that differentially expressed miRNAs were enriched nearby imprinted genomic regions other than randomly being dispersed, a permutation test was performed (Section 4). 1000 sets of miRNAs with the same quantity as the differentially expressed miRNAs were randomly generated and miRNA enrichment regions of each set were clustered using the same algorithm. The number of imprinted genes that fall in or near the miRNA clusters of each random set was, respectively, calculated and compared to 30 imprinted genes which locate in differentially expressed miRNA clusters. The results show that differentially expressed miRNAs significantly enriched near the imprinted genes (*P* = 0.037).

**Figure 2:**
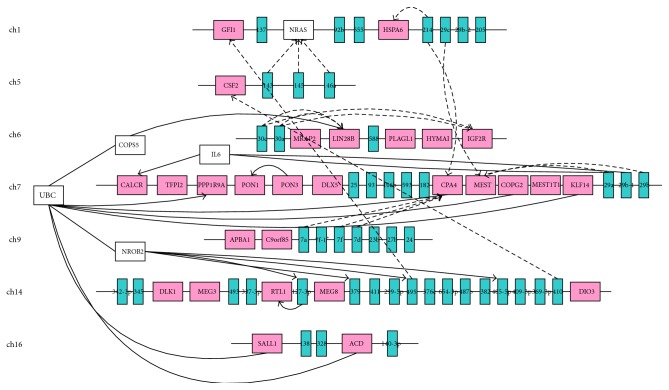
miRNA cluster regions and imprinted genes on chromosomes. Location of miRNA (blue) cluster regions and imprinted genes (pink) on chromosome, as well as the interaction between miRNAs and their target genes. The solid lines represent the interaction through IPA database, while the dotted lines represent the predicted target genes of miRNAs by both miRanda and TargetScan.

We then focus on miRNA enrichment region on chromosomes 1, 5, 6, 7, 9, 14, and 16 including most of the miRNAs (blue) and imprinted genes (pink) (Figure 2). Lines represent the pairwise interaction, while dashed lines represent the predicted target gene of miRNA. The genes in white are the predicted target genes other than imprinted genes. miRNA-143, miRNA-145, and miRNA-146a located on chromosome 5 have the same target gene N-ras, which is one of the Ras gene family members located on chromosome 1. N-ras functions as an important factor in a large number of biological process involving cell cycle progression, differentiation, cell proliferation, apoptosis, and cell survival by the level of Ras expression [24–26]. Besides, N-ras has induced tumorigenesis through different signalling pathways [27, 28]. miRNA-7a, miRNA-7f, and miRNA-7d clustering on chromosome 9 have the same target gene CPA4/CASP8, which was originally identified as an initiator caspase and mainly functions in the death receptor pathway of apoptosis [29, 30]. Many studies also explored the significance of the nonapoptotic function of caspase-8 and its mechanism [31, 32]. miRNA-29a, miRNA-29b-1, and miRNA-29b-2 clustering on chromosome 7 have the same target gene MEST/PEG1, loss of which will cause intrauterine growth retardation and abnormal maternal [33]. Both of the CPA4 and MEST are imprinted genes and located close on chromosome. Besides, polyubiquitin gene UBC interacted with many of the target indirectly and directly. Disruption of UBC reduces the absolute number of hematopoietic stem cells in embryonic livers [34] and embryonic lethality with defective fetal liver development [35]. Ubiquitin was also shown to exhibit various functions of phenotypes including cell development and cell cycle progression [36, 37].

### 2.3. miRNAs Encoded in the Imprinted CPA4-KLF14 Region Associated with Cell Pluripotency Levels

**Figure 3:**
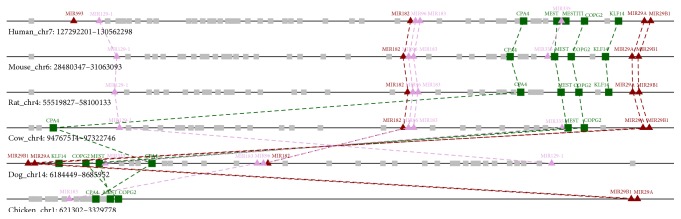
Synteny map of CPA4-KLF14 imprinted region. CHSs including CPA4-KLF14 region between human and other mammalians were constructed, respectively, and merged. The imprinted genes and miRNAs with significant regulation in hESCs or hiPSCs are shown as green rectangles and red triangles; the unaltered genes and miRNAs are shown as grey rectangles and pink triangles.

Previous studies have revealed that the Dlk1-Dio3 region in mammals will activate cell pluripotency level [8]. Here, we also find a similar imprinted CPA4-KLF14 region on chromosome 7 encoding both miRNAs and imprinted genes, which we think may have effect on the extent of cell pluripotency. In our result, the abundance of hsa-miR-29a and hsa-miR-29b decreased, while hsa-miR-593 and hsa-miR-182 increased more in iPSCs and hESCs than in NHDFs. Although mir-29a/b is reduced in both hiPSCs and hESCs, they fall further in hESCs than in hiPSCs. The inhibition of mir-29a/b may enhance cell reprogramming efficiency as previous studies have showed [38]. The distance in their expression level will directly affect the extent of cell pluripotency. This region also includes five imprinted genes, CPA4, MEST, MESTIT1, COPG2, and KLF14. Gene MEST is the predicted target gene for nearby mir-29. Furthermore, KLF14 is of the same family as KLF4, which is a well-known transcription factor and will reset the somatic cell epigenome during induced pluripotent stem cell generation [39]. Synteny map between human and other mammalians was constructed, respectively, and merged together (Figure 3). CPA4-KLF14 region locates in a genome segment which has conserved content chromosomal homologous segments (CHSs) in mouse (*P* = 1.32*E* − 207), rat (*P* = 2.32*E* − 196), cow (*P* = 4.47*E* − 193), dog (*P* = 8.69*E* − 157), and chicken (*P* = 1.15*E* − 48) (see Section 4 and Supplementary Figure 1). The gene content and order of this segment region were conserved during evolution which reflected important functional relationships between miRNAs and imprinted genes and may affect the cell fate together.

### 2.4. Interactions between Differentially Expressed miRNAs and Imprinted Genes

**Figure 4:**
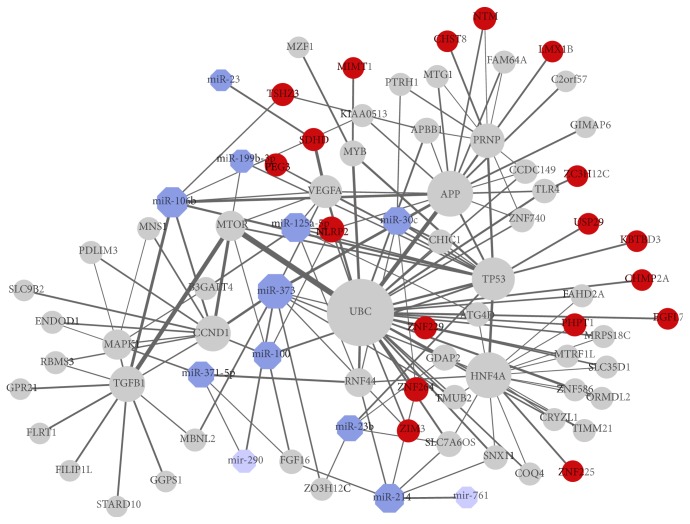
Molecular network of pluripotency related module between iPSC and ESC. Interactions of differentially expressed miRNAs (blue), imprinted genes (red), and other linked miRNAs (light blue) and genes (grey) from IPA database. Node size is proportional to the number of its links.

After viewing the global properties of the differentially expressed miRNAs between each pair of cells, we examined details of those 11 miRNAs altered significantly between iPSCs and ESCs to see whether the expression level will have effect on the pluripotency extent. Molecular network analysis was performed using IPA system to show the relationship between miRNAs and their linked genes (Figure 4). Based on topology, these differentially expressed miRNAs (blue) are connected with imprinted genes (red) closely. Diseases and function analysis showed that this network was primary centred by gene UBC, TP53, and APP and enriched in modules such as cancer, dermatological disease and conditions, organismal development, tissue development, cell death and survival, and tumor morphology (Supplementary Table 3).

Interestingly, UBC was also centred in Figure 2 and strongly interacts with several imprinted genes such as EGFL7, KBTBD3, CHMP2A, and ZC3H12C. Meanwhile, APP and TP53 are connected with UBC as well as other imprinted genes. P53 was first identified as a direct repressor of Nanog in mESCs [40]. Likewise, p53 was further proved to be functioning in apoptosis and differentiation of hESCs [41, 42] and downregulates proliferation and self-renewal of neural stem cells and hematopoietic stem cells [43, 44]. P53 is connected with miR-100, miR-30c, miR-23b, miR-125a-5p, and miR-199b-3p. APP encodes a cell surface receptor and transmembrane precursor protein amyloid beta, which decreases several cell signalling pathways associated with neurogenesis [45] and inhibits the proliferation of neural stem cells by activating the PI3K pathway [46]. These indicated that imprinted genes interact directly with pluripotency related genes.

Here, miR-373, miR-371-5p, and miR-106b show the same expression alteration but different range in hiPSCs and hESCs compared to NHDFs, which indicates that these miRNAs should be upregulated to some extent to maintain the capacity of cell pluripotency in hESCs. Researches have reported that the mmu-mir-290/hsa-mir-371/372/373 cluster expressed in trophoblast stem cells and functioned in cellular self-renewal [47, 48]. For the same reason, other miRNAs may support the cell pluripotency by different expression level. Meanwhile, other miRNAs shown in the network connected mostly with p53 may work in maintaining cell pluripotency.

## 3. Conclusions

In this study, we examined pluripotency-associated miRNA expression in human ESCs, iPSCs, and NHDFs and identified a significant correlation between miRNAs clusters within imprinted gene region. We then discuss one of the small regions on chromosome 7 which include significantly expressed miRNAs as well as four imprinted genes. The miRNAs encoded in this cluster have been verified involving cell reprogramming and proliferation and target the nearby imprinted gene MEST. Although the meaning of this relationship is not known, this imprinted CPA4-KLF14 region located in content conserved CHSs of mammals and may play important roles during transcriptional regulation and processing. Meanwhile, we construct the interaction network between differentially expressed miRNAs and imprinted genes and found that those miRNAs did interact with imprinted genes directly or indirectly through other genes. Regardless of the molecular function, the imprinted CPA4-KLF14 region observed in our research may provide a new look for those interested in different pluripotent level between hiPSCs and hESCs.

## 4. Methods

### 4.1. Transcriptome Profiles Analysis

Microarray dataset GSE16654 for 1 NDF line, 5 iPSC lines, and 3 ESC lines was obtained from GEO (http://www.ncbi.nlm.nih.gov/geo/). Each sample has three biological replicates. As discussed in [49], iPSCs were generated from NDFs by ectopic expression of the defined transcription factors KLF4, OCT4, SOX2, and C-MYC, while the HESCs were obtained from the inner cell mass of a human preimplantation embryo. These various iPSCs resources such as virus-integrating iPSCs, vector-free iPSCs, and protein-directed reprogramming iPSCs show similar biological properties to human ESCs and fall into the same iPSCs category in our study. Routine analysis including normalization and statistical difference was performed using R package. Differentially expressed miRNAs were identified between pairwise cell types by linear model.

### 4.2. Identifying Genomic Clustered Region

PTMCluster [23] was first developed to find statistically significant PTM site clusters on the same protein using a positional distance tree. Here, in order to define clustered miRNA regions, we adjust the algorithm and search the differentially expressed miRNAs on chromosomes and a *p* value *P* is calculated to evaluate whether miRNAs in the cluster are close enough in space than being randomly distributed,

(1) $$\begin{array}{c} P={\left(1-{\left(1-\frac{M}{L}\right)}^{D}\right)}^{N}. \end{array}$$

Here, *L* is chromosome length. *M* is the number of miRNAs on the whole chromosome. *D* is the maximum distance between neighbour miRNAs in the cluster region. *N* is the number of differentially expressed miRNAs in the cluster region. Significant clustered region will be selected if it satisfies the *p* value and site number cutoffs as the tool recommended (*P* ≤ 0.1; *N* ≥ 3).

### 4.3. Permutation Test

To find the physical location between those miRNAs and imprinted genes, all imprinted genes were collected from Geneimprint (http://www.geneimprint.com/). We scan the total 210 human imprinted genes on chromosome and found that 30 fall in or nearby (at most 6 Mbp) our 12 miRNA clusters. A permutation test was then performed to verify whether the imprinted genes were enriched near those pluripotency related miRNAs other than appear nearby randomly. In this test, 1000 sets of miRNAs with the same quantity as the differentially expressed miRNAs were randomly selected from 800 miRNA in profile. Each set of miRNAs were then clustered by PTMCluster algorithm and the number of imprinted genes that fall in or nearby the miRNA clusters of each random set was, respectively, calculated by the same criteria. Only 37 permutated tests (out of 1000 tests) have more than 30 imprinted genes that fall in or nearby miRNA clusters, which indicated that the imprinted genes were enriched near differentially expressed miRNA clusters in our study (*P* = 0.037).

### 4.4. Molecular Network Analysis

Target genes for known miRNA were predicted through both miRanda (http://www.microrna.org/microrna/home.do) and TargetScan (http://www.targetscan.org/). Intersection part of the results was considered as the target genes for miRNAs. Ingenuity pathway analysis (IPA) system (Ingenuity Systems, http://www.ingenuity.com/) was used for molecular networks and disease module enrichment. It collects data from a variety of experimental platforms at multiple levels and provides a comprehensive pathway resource including information from literature, gene expression, and gene annotation. Fisher's exact test was implemented to determine whether a disease or function module is enriched with genes of interest. IPA helps us to provide insight into the molecular and chemical interactions, cellular phenotypes, and disease processes of our own interested molecules and construct the related pluripotent network.

### 4.5. Synteny Map Construction

We used CHSMiner [50] to construct synteny map for human and other mammalians. It detects CHS between two genomes based on shared gene content alone and gives each pair CHS a *p* value to reflect the probability that a given CHS is observed in two independently and randomly ordered genomes. The CHSs including the CPA4-KLF14 region between human and mouse, rat, cow, dog, and chicken were identified, respectively, and shown in Supplementary Figure 1.

## Supplementary Material

Supplementary Figure 1: Synteny maps of CPA4-KLF14 region between human and other mammalians.
Supplementary Table 1: 79 differentially expressed miRNAs with at least 2 fold change.
Supplementary Table 2: GO and KEGG enrichment results.
Supplementary Table 3: Diseases and function analysis.
