# Supplementary figures and images for: Cell Pluripotency Levels Associated with Imprinted Genes in Human
Source: Comput Math Methods Med. 2015 Oct 4;2015:471076. doi: 10.1155/2015/471076 (PMC4609408; doi:10.1155/2015/471076)

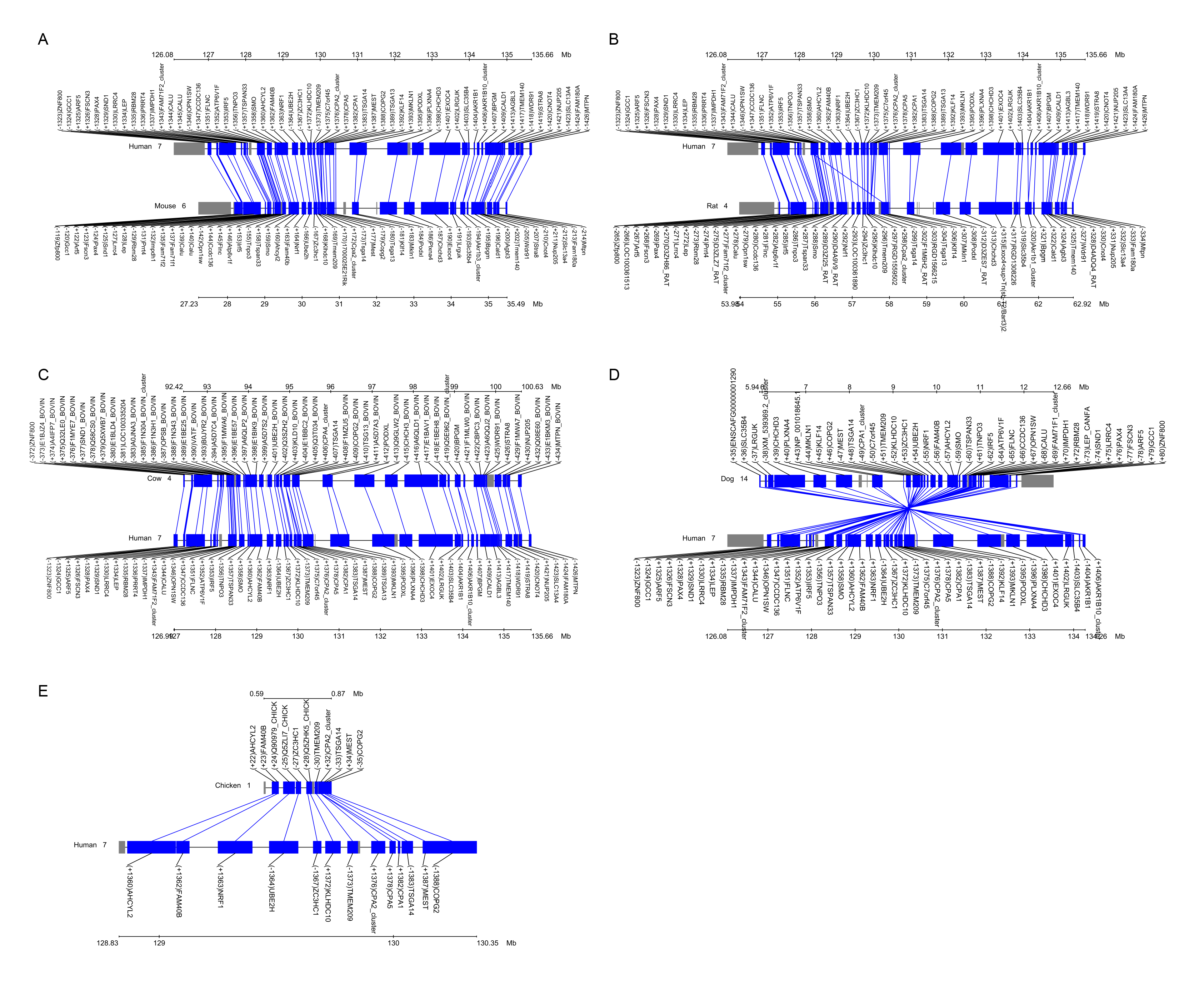

Supplement: Supplementary file 1 — Supplementary Figure 1: Synteny maps of CPA4-KLF14 region between human and other mammalians. Supplementary Table 1: 79 differentially expressed miRNAs with at least 2 fold change. Supplementary Table 2: GO and KEGG enrichment results. Supplementary Table 3: Diseases and function analysis. [file 471076.f1.zip › Supplementary Figure1.png]
